# Antioxidant Properties and Protective Effects of Some Species of the Annonaceae, Lamiaceae, and Geraniaceae Families against Neuronal Damage Induced by Excitotoxicity and Cerebral Ischemia

**DOI:** 10.3390/antiox9030253

**Published:** 2020-03-20

**Authors:** Narayana Pineda-Ramírez, Fernando Calzada, Iván Alquisiras-Burgos, Omar Noel Medina-Campos, José Pedraza-Chaverri, Alma Ortiz-Plata, Enrique Pinzón Estrada, Ismael Torres, Penélope Aguilera

**Affiliations:** 1 Laboratorio de Patología Vascular Cerebral, Instituto Nacional de Neurología y Neurocirugía “Manuel Velasco Suárez”, México CDMX 14269, Mexico; narayana_pinedar@yahoo.com.mx (N.P.-R.); burgos_inc@hotmail.com (I.A.-B.); 2 Unidad de Investigación Médica en Farmacología, Hospital de Especialidades, 2 piso CORSE, Centro Médico Nacional Siglo XXI, IMSS, México CDMX 06725, Mexico; fercalber10@gmail.com; 3 Departamento de Biología, Facultad de Química, Universidad Nacional Autónoma de México, México CDMX 04510, Mexico; omarnoelmedina@gmail.com (O.N.M.-C.); pedraza@unam.mx (J.P.-C.); 4 Laboratorio de Neuropatología Experimental. Instituto Nacional de Neurología y Neurocirugía “Manuel Velasco Suárez”, México CDMX 14269, Mexico; aortizplata@yahoo.com.mx; 5 Unidad del Bioterio, Facultad de Medicina, Universidad Nacional Autónoma de México, México CDMX 04510, Mexico; epinzone@unam.mx (E.P.E.); ismael.torres10@hotmail.com (I.T.)

**Keywords:** antioxidants, excitotoxicity, stroke, Annonaceae, Geraniaceae, Lamiaceae

## Abstract

This study aimed to compare the antioxidant activities of extracts obtained from three plant families and evaluate their therapeutic effect on strokes. Ethanol extracts were obtained from either the leaf or the aerial parts of plants of the families Annonaceae (*Annona cherimola, A. diversifolia, A. muricata, A. purpurea,* and *A. reticulata*), Lamiaceae (*Salvia amaríssima* and *S. polystachya*), and Geraniaceae (*Geranium niveum* and *G. mexicanum*). Extracts were analyzed in terms of hydroxyl radical (OH•), peroxyl radical (ROO•), and superoxide anion (O_2_•^−^). The efficiency of the extracts to prevent neuronal death induced by excitotoxicity was tested with the tetrazolium assay, the O_2_•^−^ scavenging capacity was evaluated with the dihydroethidium dye, and the protective effect of the extracts with the highest antioxidant activity was tested on a stroke experimental model. The extracts’ IC_50_ values (μg/mL) of scavenging varied from 98.9 to 155.04, 4.5 to 102.4, and 20.2 to 118.97 for OH•, ROO•, and O_2_•^−^, respectively. In the excitotoxicity model, Annonaceae extracts were highly cytotoxic while Lamiaceae and Geraniaceae reduced intracellular O_2_•^−^ production and protect neurons against oxidative stress. *Salvia polystachya* reduced cerebral damage, as well as improved survival and behavior after ischemia. Our results encouraged the use of plant extracts as natural antioxidants to minimize neuronal injury following stroke.

## 1. Introduction

Obstruction of a main cerebral artery reduces the blood flow to the brain and causes cerebral ischemia, as occurs in a stroke. As a consequence, limited glucose and oxygen supply lead to neuronal damage. Initially, injury results from inhibition of oxidative phosphorylation that decreases adenosine triphosphate (ATP) production that, among other changes, provokes inactivation of the N^+^/K^+^-ATPase and an excessive release of glutamate. The over-activation of glutamate receptors induces calcium accumulation at the mitochondria level, favoring free radical production by the mitochondrial respiratory chain [1]. The generation of oxidative stress is a crucial point for the induction of neuronal injury observed after cerebral ischemia; therefore, treatment with antioxidants has shown a protective effect through different mechanisms of action in experimental models [2]. However, the characterization of other antioxidant compounds is still required since results obtained with the current ones indicate these are not suitable yet.

In the past years, several extracts of plants have appeared on the market as antioxidants, and the physiological activities of the natural components have been identified. Interestingly, many food sources potentially have beneficial effects in counteracting cardiovascular complications, such as coronary heart disease and stroke [3]. The antioxidant capacity of some of these mixtures showed comparable or even higher activities than synthetic antioxidants [4,5]. Moreover, synthetic antioxidants frequently have toxic and other undesirable side effects. Thus, the characterization of innocuous, safer, and low-cost effective antioxidants from natural sources, such as vegetables and plants, is still necessary.

In this line, resveratrol (RSV), a stilbene found in grapes (among many other plant products), has been widely recognized for its ability to modulate the dynamics of the cellular redox-status in vitro and in vivo in experimental models of ischemia [2,6]. The beneficial effects of RSV in ischemia include the suppression of production of superoxide anion (O_2_•^−^), providing neuroprotection through regulation of pathways sensitive to oxidative stress, such as AMPK/SIRT and PI3K/AKT/mTOR [7]. Recently, it was reported that in cerebral ischemia and in a model of oxygen and glucose deprivation, RSV modulates autophagy, the process that removes and recycles damaged cellular components through AMPK activation [8]. Because RSV is in the spotlight due to its effects on the human body suffering from disease and its low toxicity [9,10], we used it as a reference in the neuroprotective effect against ischemia-induced damage in vitro and in vivo. 

Several species of medicinal plants have neuroprotective activities for the CNS. These effects are mainly associated with their composition in antioxidant compounds such as flavonoids and terpenoids. Plants of the family Lamiaceae, Annonaceae, and Geraniaceae have been traditionally used to treat central nervous system pathological conditions in several models, including cerebral ischemia [11,12,13]. Therefore, we include plants of these families in our study.

Annonaceae is a large family of plants consisting of about 2300 to 2500 species, and more than 130 genera distributed mainly in tropical and sub-tropical regions. The presence of alkaloids, flavonoids, and acetogenins found in leaves, bark, seeds, and fruit is a chemical distinctive of this family. Concerning the species of Annonaceae, those from the genus *Annona* comprise approximately 175 species of trees and shrubs. In Mexico, the genus *Annona* contains around 60 species in 12 genera, with the largest number found in forests. Among these, the most cultivated are *Annona cherimola, A. muricata, A. reticulata, A. diversifolia, A. purpurea*, and *A. squamosa*. Furthermore, economically, this genus is the most appreciated of the Annonaceae family due to its edible fruits, nutritional value, and medicinal properties [14]. Annonaceae inhibit production of O_2_•^−^ production [15]. O_2_•^−^ is the primary and more damaging reactive oxygen specie produced during cerebral ischemia and recovery of blood flow (reperfusion) [16]. The extract obtained from *A. diversifolia* also reduced the severity of behavioral and electroencephalographic seizures, supporting its effect on the central nervous system [17]. 

Geraniaceae is a family comprising about 11 genera and more than 730 species widely distributed in subtropical, tropical, and temperate areas worldwide. In the case of the genus *Geranium*, it constitutes around 300 species. Some of these species are high multi-harvest species, with high value as aromatic cultivated herbs for their essential oils, which are widely used in the cosmetic industry and to flavor foods [18]. In Mexico, the genus *Geranium* grows along the dry stream banks and grassy meadows of the pine–oak forests, and of this genus the species *Geranium niveum* and *G. mexicanum* are used as medicinal plants or ornamentals [19]. Several reports indicate the antiprotozoal activity of these plants as their bioactivity due to compounds such as flavonoids [20]. *G. mexicanum* exhibits a high content of cathechin [21], an active compound that shows neuroprotective effects against cerebral ischemia [22]. Additionally, cathechin protect against cell apoptosis in myocardial ischemia and reperfusion injury, supporting its beneficial effects by modulating pathways sensitive to oxidative stress [23]. 

The Lamiaceae family includes nearly 1000 species organized in four sections: Salvia, Leonia, Clarea, and Calosphace [24]. *Salvia* is the principal genus in the mint family; these plants are shrubs, herbaceous perennials, and annuals. Many *Salvia* species are used in cooking as herbal tea and for food flavoring. They are also used in cosmetics, perfumery, and pharmaceutical industries around the world [25]. Importantly, many species of Lamiaceae have been used traditionally for the treatment of a variety of neurodegenerative disorders and some have shown protective properties in cerebral ischemia, reducing lipid peroxidation, restoring the glutathione content, and attenuating motor impairment [12]. In Mexico the *Salvia* species are included in the Mirto complex; it includes *Salvia polystachya* and *S. amarissima*. The bioactive clerodane diterpenes are abundant in both species [26]. Clerodanes can be used to activate opioid receptors and protect against cerebral ischemia [27]. 

Our main objective was to characterize the antioxidant capability of extracts obtained from various plants from Annonaceae, Lamiaceae, and Geraniaceae families. We examined the hydroxyl radical (OH•), peroxyl radical (ROO•), and O_2_•^−^ scavenging capacity of nine extracts using either the leaves or aerial parts of the plants. Besides, we tested the efficiency of the extracts to prevent the neuronal death induced by excitotoxicity, and their in vitro O_2_•^−^ scavenging activity. Finally, we evaluated the protective effect of two plant extracts (with the highest scavenging activity for O_2_•^−^) in a focal cerebral ischemia model in vivo. Results support the high antioxidant capacity of these extracts and their ability to protect neurons against oxidative stress produced during injury. 

## 2. Materials and Methods 

### 2.1. Materials

GlutaMAX™ (35050-061), basic fibroblast growth factor β (BFGFβ), Hoechst 33342 (H1399), (DMEM, 12800-017), neurobasal medium (21103049), and B-27 supplement (17504-044) were purchased from Thermo Fisher Scientific (Waltham, MA, USA). Poly-L-lysine (P1524), cytosine-β-D-arabino furanoside (C1768), L-glutamate (G2834), glycine (D8898), 3-(4, 5-dimethylthiazol-2-y)-2, 5-diphenyltetrazolium bromide tetrazolium salt (MTT, M5655), dihydroethidium (DHE, D7008), and resveratrol (RSV, R5010) were from Sigma-Aldrich (St. Louis, MO, USA). Gentamycin and isoflurane were from PiSA Laboratories (CDMX, Mexico). Dimethyl sulfoxide (DMSO, 07001) was from Fermont (Monterrey, NL, Mexico). Primary antibody anti-Microtubule-Associated Protein 2 (MAP-2, AB5392) was from Abcam (Cambridge, UK). The secondary antibody Alexa Fluor^®^ 594-conjugated anti-chicken IgG (703-585-155) was from Jackson ImmunoResearch Laboratories Inc. (West Grove, PA, USA). 2, 3, 5-triphenyltetrazolium chloride (TTC, 22631) was from USB Corporation (Cleveland, OH, USA).

### 2.2. Plant Material

A list of nine plants used in traditional Mexican medicine was elaborated based on the information obtained through data-bank [28]. The plants were obtained from the field in six different states of the country: Mexico City, State of Mexico, Guerrero, Chihuahua, Chiapas, and Veracruz. Reference vouchers of the plant material were deposited at the Instituto Mexicano del Seguro Social (IMSS) herbarium (IMSSM). Plant species, botanical name, family, voucher specimens, and plant parts used to obtain the extracts are summarized in Table 1.

### 2.3. Preparation of the Plant Extracts

For each part of the plant, the ethanol extract was prepared by macerating 20 g of powdered dry plant material in stoppered flasks containing 300 mL of ethanol for 1 week (twice) at room temperature. After filtration, the solvent was evaporated under reduced pressure in a rotary evaporator. The different extracts were conserved in tightly sealed glass vials. The yields are shown in Table 1.

### 2.4. OH• Scavenging Assay 

To measure the antioxidant capacity of the extracts, OH• was generated by the reaction between Fe³⁺–ethylenediamine tetraacetic acid (EDTA) and hydrogen-peroxide (H₂O₂). The generation of the radical was assessed using terephtalic acid (TA) since the non-fluorescent compound TA reacts with OH• to form fluorescent 2-hydroxy-TA [29]. The reaction was a mix of the following compounds in 160 µL: 0.2 mM ascorbic acid, 0.2 mM FeCl_3_, 0.208 mM EDTA, and 1.4 mM TA in 20 mM phosphate buffer (pH 7.4), and were mixed with 20 μL of distilled water (0% scavenging tube) or with 20 μL of the different concentrations of samples. The reaction started with the addition of 1 mM H₂O₂ (20 μL). The fluorescence signal was measured for 30 min at a wavelength of excitation of 326 nm and emission of 432 nm in a Synergy™ HT Multi-Mode Microplate Reader (BioTek Instruments, Inc., Winooski, VT, USA). The OH• scavenging abilities were interpolated to obtain the 50% inhibitory concentrations (IC_50_). RSV was used as standard OH• scavenger.

### 2.5. ROO• Scavenging Assay

The assays were based on the Oxygen Radical Absorbance Capacity (ORAC) test [30], which is a method that measures the antioxidant capacity of a substance. The ORAC assay measures a fluorescent signal from a probe that is quenched in the presence of reactive oxygen species (ROS). The addition of an antioxidant absorbs the generated ROS, allowing the fluorescent signal to persist. We use 2, 2’-Azobis (2-methylpropionamidine) dihydrochloride (AAPH), a water-soluble azo compound, as a ROO• generator; Trolox^®^ (6-hydroxy-2,5,7,8-tetramethylchromane-2-carboxylic acid, St. Louis, MO, USA), a water-soluble vitamin E analog and a well-known antioxidant, was used as the standard; and fluorescein as a fluorescent probe. The ORAC test produces a ROO• free radical upon thermal decomposition. This assay depends on the peroxyl radical damage to fluorescein molecules that results in a loss of fluorescence and when an antioxidant is present its antioxidant capacity correlates to the fluorescence decay curve, which is usually represented as the area under the curve. Briefly, the assay was carried out as follows: 25 μL of water, Trolox^®^ as the standard, or diluted samples or diluted vehicles were mixed with 25 μL of 153 mM AAPH and with 150 μL of 50 nM fluorescein and incubated at 37 °C. The fluorescence was measured every minute for 90 min using fluorescence filters for an excitation wavelength of 485 nm and an emission wavelength of 520 nm. 

### 2.6. O₂•^−^ Scavenging Assay

The xanthine–xanthine oxidase (XO) system was used to generate O_2_•^−^ [31]. This enzymatic system is useful to test for O_2_•^−^ scavenging capacity. XO catalyzed the oxidation of hypoxanthine to xanthine and subsequently to uric acid. During the re-oxidation of XO, molecular oxygen acts as an electron acceptor. During these reactions, O_2_•^−^ radicals are formed. To avoid false positives, it was necessary to prove that the sample does not inhibit XO activity by measuring uric acid production. O_2_•^−^ generation and XO activity were measured as nitroblue tetrazolium (NBT) reduction and uric acid production, respectively. The assay was made as follows: 160 μL of the following reaction mixture (90 μM xanthine, 16 mM Na_2_CO_3_, 22.8 μM NBT, and 18 mM phosphate buffer (pH 7.0)) were mixed with 20 μL of distilled water (0% scavenging tube) or with 20 μL of different concentrations of the samples. The reaction was initiated by the addition of 20 μL of xanthine oxidase (168 U/L). Optical density was registered both at 295 nm (for uric acid production) and at 560 nm (for O_2_•^−^ generation). RSV was used as standard O_2_•^−^ scavenger.

### 2.7. Experimental Design

Ischemic damage was induced with the middle cerebral artery occlusion (MCAO) model in rats. Rats were randomly distributed in 3 groups (*n* = 10): CT, sham animals, subjected to surgical procedure without induced ischemia; +Vh, ischemic rats plus vehicle; and +Sp, ischemic rats plus Sp treatment. Neuronal primary cultures of the Wistar rat brain cortex were used to analyze the effect of plant extract and RSV on cellular damage. Cultures were exposed to excitotoxicity (10 min), followed by 30 min, 2 h, or 24 h of recovery (change of medium). Cultures were divided into 2 main groups (*n* = 4): (1) CT, cells to which the culture medium was changed by Krebs–Henseleit (KHB) solution followed by recovery; and (2) Glu, cells exposed to excitotoxicity followed by recovery. Treatments were added to the cells during the recuperation.

### 2.8. Treatment with Plant Extracts 

All stock plant extracts were dissolved in dimethyl sulfoxide (DMSO) at 1 mg/mL. In vitro *excitotoxicity model*: plant extracts (0.00001 to 1 μg/μL) dissolved in DMEM-B27 were added to cell cultures after excitotoxicity treatment and were maintained during the recuperation period (24 h). The final concentration of DMSO during the treatment was 0.1%. RSV (dissolved in 0.1% ethanol) and was used in a concentration range from 0.004 to 400 µM. In vivo *ischemia model*: animals were injected at the onset of reperfusion in the tail vein with 100 μL of either plant extract (3 mg/kg) or vehicle (50% ethanol in double-distilled H₂O). The dose was chosen according to previous experiments with plant extracts with protective effects. 

### 2.9. Oxidative Stress in Primary Neuronal Cultures Induced by Excitotoxicity

Primary cortical neuronal cultures were prepared as described previously [32] with some modifications. The cerebral cortex was obtained from 18-day-old Wistar rat embryos. Cells were plated on poly-L-lysine coated plates seeded at 25 × 10^3^ cells/cm^2^ for immunofluorescence experiments and 50 × 10^3^ cells/cm^2^ for the cytotoxicity experiments and maintained at 37 °C in a humidified atmosphere of 5% CO_2_/95% air on Neurobasal medium supplement with B-27 1X, serum-free, GlutaMAX™ 1X, 5 ng/mL of basic fibroblast growth factor β, and 1.26 mg/mL of gentamicin. On Day 4, a change of 50% of the medium was performed adding 4 μM cytosine-β-D-arabino furanoside (AraC) to inhibit glial growth. Neuronal cultures were used 8 days after being seeded. Excitotoxicity was induced by treatment with 100 µM _L_-glutamate and 10 µM glycine for 10 min.

### 2.10. Determination of Cytotoxicity 

Cytotoxicity was tested using the 3-(4, 5-dimethylthiazol-2-y)-2, 5-diphenyltetrazolium bromide tetrazolium (MTT) assay. After 24 h of treatment with the extract, the medium was recollected and the cultures were incubated with 0.5 mg/mL of MTT solution (37 °C, 4 h in the darkness). Formazan crystals were solubilized adding 100 µL of DMSO. Absorbance was measured at 570 and 690 nm. Results were expressed as the percent MTT reduction relative to the control. 

### 2.11. Immunofluorescence to Identify Neurons

Cells were fixed with 0.5% formaldehyde and permeabilized with methanol. Nuclei were stained with 10 μg/mL Hoechst 33342 and an anti-MAP-2 antibody, followed by Alexa Fluor^®^ 594-conjugated anti-chicken IgG used to identify neurons (1:10,000) by fluorescence microscopy (20X dry objective, with an Olympus model 1 × 71 microscope (Olympus Corporation of the Americas, Center Valley, PA, USA). Immuno-stained assays for glial fibrillary acidic protein revealed that the presence of astrocytes in neuronal cultures was <98%.

### 2.12. Measurement of Intracellular O₂•^−^ Production Induced by Excitotoxicity 

Cells were stimulated for 30 min (during recovery) with the extract (0.01 μg/μL Sp, 0.001 μg/μL GmPA, and 0.0001 μg/μL GnPA and Sa). After, cells were incubated with 0.02 mmol/L dihydroethidium dye (37 °C, 2 h in the darkness). The fluorescence of complex ethidium-DNA was measured at an excitation wavelength of 480 nm and an emission wavelength of 610nm. To normalize the fluorescence intensity, the total amount of protein was measured with the bicinchoninic acid assay.

### 2.13. Induction of Cerebral Ischemia with the Middle Cerebral Artery Occlusion (MCAO) Model

Handling of animals was executed under the internal protocol approved by the Institutional Animal Care and Use Committee of the Instituto Nacional de Neurología y Neurocirugía “Manuel Velasco Suárez” and in accordance to the NIH Guide for the Care and Use of Laboratory Animals according to NOM-062-200 and the World Medical Association Declaration of Helsinki for the animal use in biomedical research. Transient focal cerebral ischemia was induced with the MCAO method previously described [33]. Briefly, male Wistar rats (280–350 g) were anesthetized with a mixture of 2% oxygen and 2.5% isoflurane and then maintained at 37 °C. The surgical procedure consisted of blocking cerebral blood flow into the MCA territory by introducing a nylon monofilament suture 3-0 into the left internal carotid artery. After 2 h occlusion, animals were newly anesthetized, and the filament was removed to allow the restoration of the MCA blood flow (reperfusion). The neurological deficit was evaluated during MCAO with two behavioral tests: rats that fail to extend the right forepaw fully and accomplish turning to the left (≥5 rounds/1 min) were included in the protocol. Reperfusion was allowed for 24 h; then animals were sacrificed.

### 2.14. Quantification of Tissue Damage, Neurological Outcome, and Survival after MCAO

Injury-induced by ischemia was measured using the TTC salt staining. Rats were sacrificed after 2 h MCAO and 24 h reperfusion. Then, brains were removed and frozen (5 min, −80 °C). Serial coronal slides (2.5 mm width) were obtained and stained with 2% TTC in the darkness for 30 min at 37 °C and after were placed in 4% paraformaldehyde for 1 h. Finally, the slices were photographed with a digital camera (PowerShot S100, Canon, Japan). The percentage of the damage (infarct area) was calculated considering the total cerebral area obtained from the sum of all slices, which was considered as 100%. Image analysis was performed using Image J software [34].

The “limb-use asymmetry test” was performed 24 h after MCAO to evaluate the neurological impairment of animals. Rats were placed in a transparent acrylic cylinder, and a video was recorded in the afternoon for 5 min. Locomotor asymmetry was evaluated by a test that counts the contacts that animals make with their extremities on a cylinder wall [35]. The global use score of extremities (BIAS) was calculated by subtracting the percentage of contacts of the altered to the percentage of the not altered limb. To evaluate survival, the percentage of animals that survive 24 h relative to the total number of individuals in each group was obtained (*n* = 11).

### 2.15. Statistical Analysis 

Differences between groups were analyzed by the Student’s *t*-test and one-way analysis of variance (ANOVA) with a Tukey’s test. A *p* < 0.05 was considered statistically significant. Statistical analysis was performed using GraphPad Prism 7 (GraphPad Software, San Diego, CA, USA).

## 3. Results

### 3.1. OH• Scavenging Activity of Annonaceae, Lamiaceae, and Geraniaceae Extracts 

The plant extracts showed a dose-dependent OH• scavenging activity. Among the extracts, Amu had the highest activity, with an IC_50_ of 98.96 ± 19.58 μg/mL; it was almost 3.3 times higher than the observed for RSV (IC_50_ = 30.02 ± 6.58 μg/mL). The rest of the extracts had an antioxidant activity with an IC_50_ varied from 126.98 ± 31.60 to 155.04 ± 4.40; showing that all the samples analyzed had similar OH• scavenging values (Table 2). 

### 3.2. ROO• Scavenging Activity of Annonaceae, Lamiaceae, and Geraniaceae Extracts 

The ROO• scavenging activity for the extracts was dose-dependent. RSV had an IC_50_ = 0.23 ± 0.06 μg/mL for ROO•. Regarding plant extracts, Ap had the highest capacity with an IC_50_ = 4.49 ± 0.24 μg/mL whereas the other extracts exhibited IC_50_ values ranging from 10.46 ± 2.47 to 30.42 ± 8.0 μg/mL and Ach with the highest value of 102.43 ± 42.92 μg/mL. The IC_50_ values vary independently on the plant family from which the extract was obtained (Table 2). 

### 3.3. O₂•^−^ Scavenging Activity of Annonaceae, Lamiaceae, and Geraniaceae Extracts 

For the extracts Ach, Ad, Amu, and Are from the Annonaceae family, the 50% trapping of the radical was not achieved even with 3 mg/mL, the highest dose used. For the other extracts, the O_2_•^−^ scavenging activity was dose-dependent. The IC_50_ values obtained with the xanthine–xanthine oxidase method indicate that the efficacy of the extracts to neutralize O_2_•^−^ is as follows: GnPA < Ap < GmPA < Sa < Sp (Table 2). Because the antioxidants can affect the O_2_•^−^ production either by trapping or by neutralizing the radical or inhibiting the production, we also evaluated the uric acid production in the xanthine–xanthine oxidase system. Only RSV had an effect inhibiting 50% of the production of uric acid with an IC_50_ = 24.95 ± 10.12 μg/mL. 

### 3.4. Plant Extracts Obtained from Geraniaceae and Lamiaceae Families Protect Neurons from Excitotoxicity-Induced Oxidative Stress

**Figure 1:**
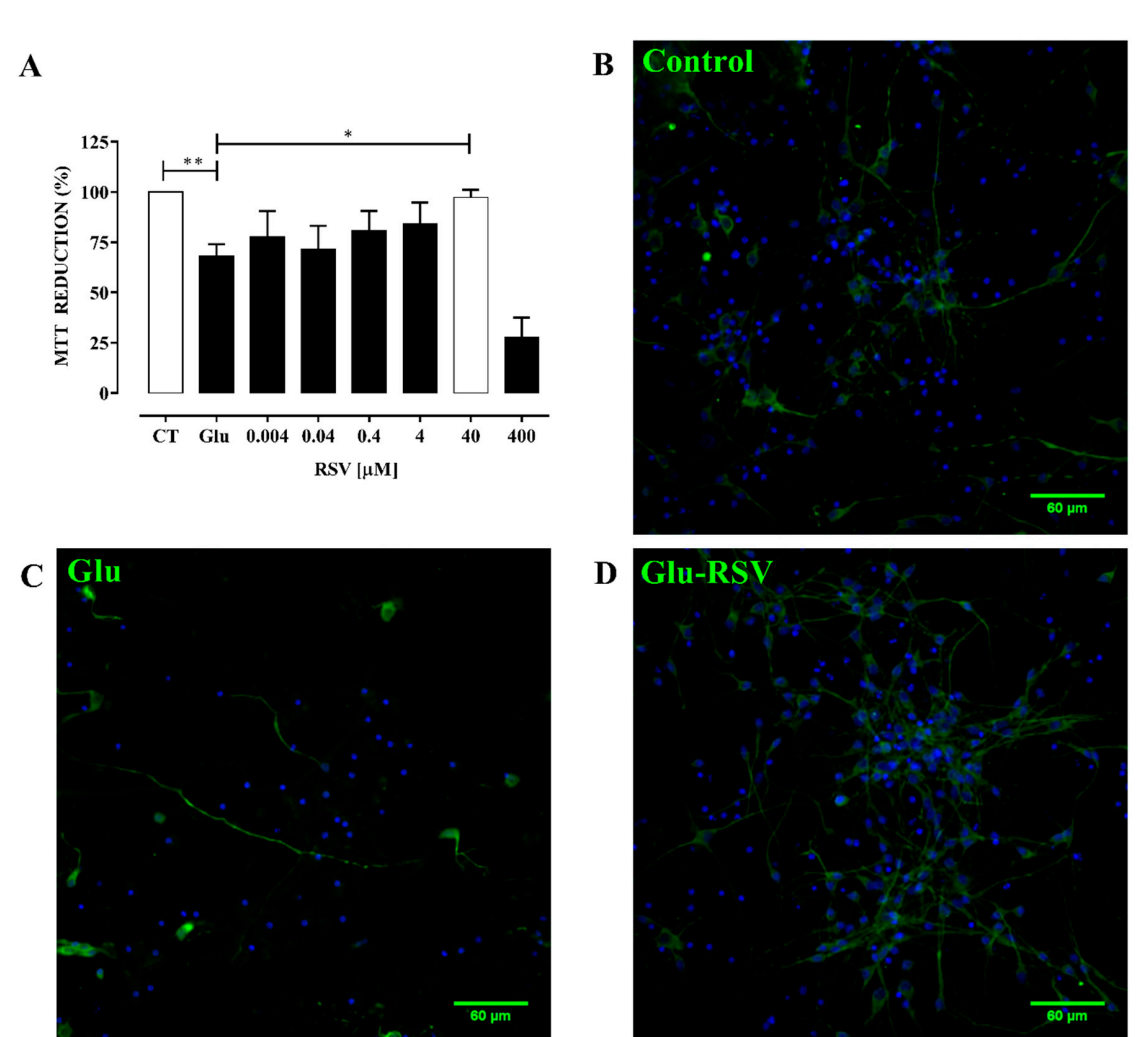
Resveratrol (RSV) protects against damage induced by excitotoxicity in cultured neurons. (**A**) RSV dose-response. Primary neuronal cultures were stimulated with 100 μM glutamate (Glu) for 10 min to induce excitotoxicity. RSV was diluted in ethanol (0.05%). After excitotoxicity, cultured neurons were treated with different concentrations of RSV during 24 h. CT, control cultures not stimulated with Glu. Glu, cultures subjected to excitotoxicity not treated with RSV. Percentage of viability was evaluated 24 h after excitotoxicity by 3-(4,5-dimethylthiazol-2-yl)-2,5-diphenyltetrazolium bromide (MTT) reduction. Results were expressed as mean ± SEM; ANOVA, *n* = 4. Difference was found between CT vs. Glu, ** *p* < 0.001; and Glu vs. Glu-RSV 40 μM (white bar), * *p* < 0.05. Representative images of neuronal cultures are showed in (**B**) Control. Neuronal culture under control conditions. (**C**) Glu. Neuronal culture exposed to excitotoxicity. (**D**) Glu-RSV. Culture expose to excitotoxicity and 40 μM RSV added at the onset of recovery. Condensates nuclei were detected by immunofluorescence microscopy with Hoechst 33342 (blue) and neurons were identified with the antibody anti-Microtubule-Associated Protein 2 (MAP-2, green).

First, the effect of RSV on cultured neurons exposed to excitotoxicity was tested. RSV prevented the reduction of cell viability induced by excitotoxicity. Its antioxidant activity was dose-dependent from 0.004 to 40 μM; however, cell viability at 400 μM RSV dropped to 27.67% ± 5.69%. The main effect was observed at 40 μM RSV with a protective effect of 97.33% ± 2.18% (Figure 1A). RSV prevented the drop on cellular viability and preserved the neuronal integrity (Figure 1A–D). 

**Figure 2:**
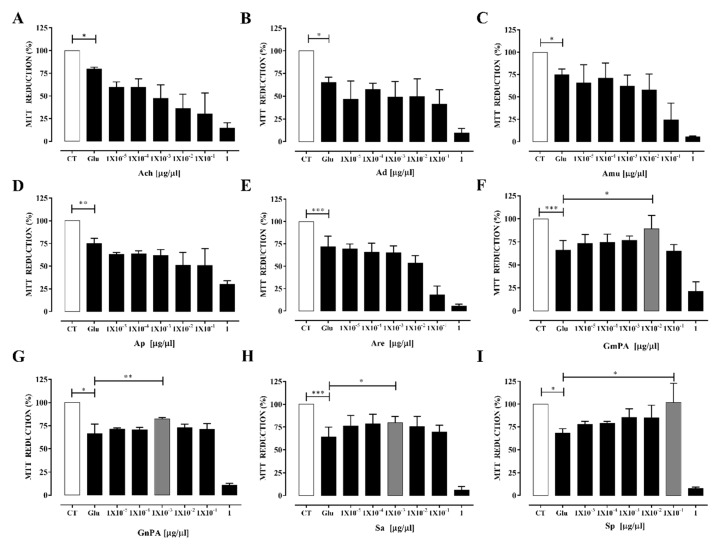
Effect of plant extracts obtained from Annonaceae, Geraniaceae, and Lamiaceae families on neuronal viability after excitotoxicity. Primary neuronal cultures were stimulated with glutamate (Glu) for 10 min to induce excitotoxicity. Different concentrations of plant extracts (0.00001–1 μg/µL) were added for 24 h after excitotoxicity. CT, control cultures not stimulated with Glu. Glu, cultures subjected to excitotoxicity not treated with plant extracts. Viability was evaluated by the 3-(4, 5-dimethylthiazol-2-y)-2, 5-diphenyltetrazolium bromide tetrazolium (MTT) reduction. The effect of the plant extract is showed in (**A**) Ach, *Annona cherimola*; (**B**) Ad, *Annona diversifolia*; (**C**) Amu, *Annona muricata*; (**D**) Ap, *Annona purpurea*; (**E**) Are, *Annona reticulata*; (**F**) GmPA, *Geranium mexicanum*; (**G**) GnPA, *Geranium niveum*; (**H**) Sa, *Salvia amaríssima*; (**I**) Sp, *Salvia polystachya*. Results are expressed as mean ± SEM; ANOVA: CT vs. Glu was significantly different. Specific concentrations of plant extract that showed differences (gray bars) against the Glu group are indicated with the upper bar. *** *p* < 0.0001; ** *p* < 0.001; * *p* < 0.05.

Then, to evaluate the antioxidant effect of the plant extracts on neuronal cultures exposed to excitotoxicity, different concentrations of the extracts were used (0.00001 to 1 μg/μL). The Annonaceae family of compounds showed either no effect or caused a decrease in neuronal viability. The most toxic extract was Ach that showed a dose-response effect. The toxicity level of the extracts belong to this family was as follows: Ach > Am > Are > Ap > Ad. However, all extracts were highly toxic to neurons at 1 μg/μL (Figure 2A–E).

Extracts from the Geraniaceae and Lamiaceae families of plants exhibited different properties: GnPA and Sa extracts showed a slight protective effect after excitotoxicity while GmPA and Sp notably prevented the damage. The maximum protective effect for GmPA was 89% ± 8.54%, observed at 0.01 μg/μL, whereas Sp showed a 100% ± 13.78% protection at 0.1 μg/μL. A cytotoxicity effect was observed for all the extracts at 1 μg/μL (Figure 2F–I).

### 3.5. Extracts Obtained from the Geraniaceae and Lamiaceae Families Reduced Intracellular Production of O₂•^−^

**Figure 3:**
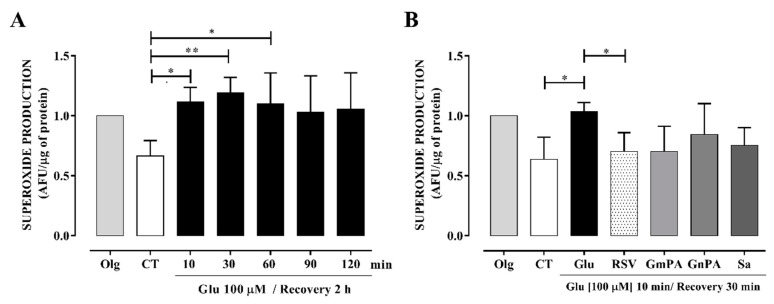
Reduction of intracellular superoxide (O_2_•^−^) levels on neuronal cultures treated with extracts obtained from the Geraniaceae and Lamiaceae families. Primary neuronal cultures were stimulated with 100 μM glutamate (Glu) to induce excitotoxicity. O_2_•^−^ production was evaluated using dihydroethidium dye. (**A**) Time course of O_2_˙^-^ production after excitotoxicity. The neuronal culture was exposed to glutamate for different periods (10, 30, 60, 90, and 120 min) and then allowed recovery for 2 h. (**B**) Effect of extracts on O_2_•^−^ levels. Neuronal cultures were exposed to 10 min of excitotoxicity and after extracts were added for 30 min. CT, control cultures not stimulated with Glu; Olg, cultures treated with oligomycin; GmPA (0.001 μg/µL), *Geranium mexicanum*; GnPA (0.0001 μg/µL), *Geranium niveum*; Sa (0.0001 μg/µL), *Salvia amaríssima*; Sp (0.01 μg/µL), and *Salvia polystachya*; Resveratrol (RSV), arbitrary fluorescence units (AFU). Results are expressed as mean ± SEM; ANOVA: Specific groups that showed differences against the CT or Glu group are indicated with the upper bar. ** *p* < 0.001, * *p* < 0.05.

We chose the less toxic extracts and tested their effect on O_2_•^−^ production induced by excitotoxicity. O_2_•^−^ production at the mitochondria was promoted by glutamate at similar levels to those produced by the mitochondrial ATP synthase inhibitor, oligomycin. This high level of O_2_•^−^ was maintained from 10 to 120 min of stimulation (Figure 3A). RSV reduced the excitotoxicity-induced O_2_•^−^ production in a 33.0% ± 6.93%. Plant extracts from Geraniaceae and Lamiaceae families reduced the O_2_•^−^ levels to similar levels than RSV. Sp and GmPA induced a reduction of 30.4% ± 8.23% and 33.15% ± 10.52% in the O_2_•^−^ production, respectively (Figure 3B). Because these compounds also protected neurons in culture, they were chosen to be tested in the in vivo model of ischemia. 

### 3.6. Sp Extract Prevented the Cerebral Ischemia-Induced Damage

**Figure 4:**
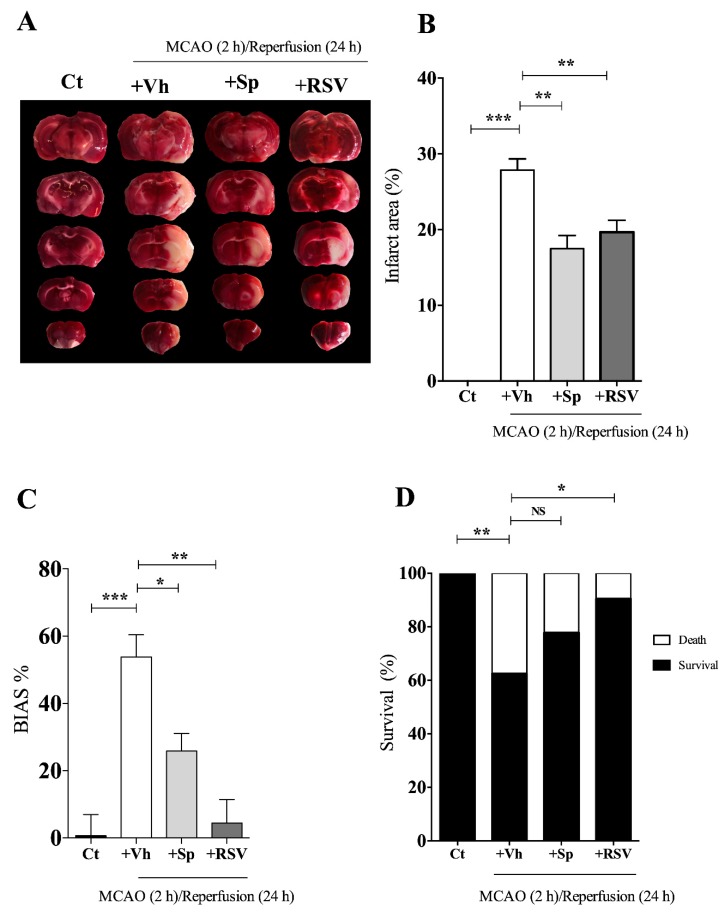
*Salvia polystachya* (Sp) extract from the Lamiaceae family prevents damage induced by cerebral ischemia. Animals were subjected to 2 h of ischemia by middle cerebral artery occlusion (MCAO) and followed by 24 h of reperfusion (*n* = 6–10). Sp extract was added at the beginning of the reperfusion (3 mg/kg; diluted in 50% ethanol; intravenous via). Control (Ct); Vehicle (+Vh); Vehicle + Sp extract (+Sp). **(A)** Effect of the treatment with Sp on the infarct area. Infarct area was evaluated in coronal brain sections by the 2, 3, 5-triphenyltetrazolium chloride (TTC) dye test. **(B)** Quantification of the infarct area. **(C)** Evaluation of neurological deficit. The global use score of extremities (BIAS) was calculated by subtracting the percentage of contacts of the altered limb to the percentage of the not altered limb. **(D)** Survival. Results were expressed as mean ± SEM; ANOVA and Tukey test: upper bars indicated the groups that present differences, *** *p* < 0.0001; ** *p* < 0.001; * *p* < 0.05. NS, non-significant.

Free radical production is mostly produced during reperfusion after MCAO [1,16]. Therefore, extracts with the higher antioxidant activity and the lower level of toxicity were administrated at the onset of reperfusion by i.v. injection. In this manner, compounds reach the brain during the first 30 min of reperfusion. The compounds’ effect was measured 24 h later. GmPA administration (3 mg/kg) caused the death of 90% of the animals, indicating a high level of toxicity of some components of the extract. We also tested the protective effect of Sp (3 mg/kg). TTC was used to calculate the infarct area. The white areas indicate infarction. We observed that the Sp-treated group showed a smaller infarct area than the MCAO group (Figure 4A,B). The effect of Sp was superior to that of resveratrol (20.76% ± 1.67%). Additionally, the Sp-treated MCAO group significantly decreased the neurological deficit compared to the Vh-treated MCAO group (Figure 4C). The resveratrol-treated group exhibited a value of 3.4% ± 7.7% on the neurological test, showing a higher protective effect that Sp. Finally, we observed that Sp prevented the decrease in survival induced after 24 h reperfusion (Figure 4D). This result also showed that Sp extract possesses important neuronal protective abilities but was not superior to resveratrol’s (90.4% ± 6.5%).

## 4. Discussion

Currently, phytochemicals are considered a promising strategy to protect cells from oxidative damage, historically because products of plant origin are essential sources of antioxidants and furthermore have low toxicity. Antioxidant therapy might prevent damage induced by free radicals in diseases that cause brain dysfunction, although definitive conclusions have not been obtained in the clinical area [36]. In the present work, we first established the free radical-scavenging activity of extracts obtained from Annonaceae, Lamiaceae, and Geraniaceae plants and assessed their effect on neuronal excitotoxicity. The antioxidant capacities of the extracts were examined with three assays: the OH• scavenging activity using the Fenton reaction, ROO• scavenging activity based on the ORAC test, and O_2_•^−^ scavenging activity by employing the xanthine–xanthine oxidase system. We selected RSV as the standard scavenger for these free radicals because RSV has a high protective effect against the cerebral ischemia-induced damage that has been associated with its electron donor properties for neutralizing free radicals [37,38] Additionally, RSV has very low toxicity [10]; however, we found that RSV 400 μM was toxic for neuronal cultures. This effect can be justified because a high concentration of RSV can exhibit pro-oxidant properties, leading to oxidative breakage of cellular DNA in the presence of transition metal ions, such as copper, and increase cellular death [39]. Such a pro-oxidant action also could be a common mechanism for anticancer and chemopreventive properties observed for some plant polyphenols [40]. 

RSV is excellent antioxidant, even though an unselective OH• scavenger [41] and all our compounds were tested against it. In our study, all the extracts tested exposed a significant OH• scavenging activity with an IC_50_ ranging from 98.96 to 154.18 μg/mL, superior to that exhibited by RSV with an IC_50_ of 30.02 μg/mL. The Amu (*Annona muricata*) extract obtained from the leaf showed the most significant activity, possibly linked to its high content of alkaloids, flavonoids, and polyphenols, which are specifically concentrated in the fruit pulp and leaf, the main storage sites of the plant [42]. The Ach (*Annona cherimola*) extract, also obtained from the leaves, showed an IC_50_ = 155.04 μg/mL, which might also be associated with its content of phenolic compounds [42]. Regardless of the family to which the other extracts belong, any appreciable difference among their ability to quench OH• was observed. Because neuronal mitochondria generate large amounts of OH•, the extracts with high activity to quench OH• could be a marker for its application in brain protection [43].

We also measured the ROO• scavenging activity because of its role in the chain-propagation mechanism of lipid peroxidation and its capacity to diffuse to remote cellular loci. RSV acts as an efficient ROO• scavenger (IC_50_ = 0.23 ± 0.06). The values of the extracts ranged from 4.48 ± 0.24 for Ap to 102.43 ± 42.92 for Ach. These results show that the ROO• scavenging activity of the extracts is lower compared to our reference compound (RSV). We observed that Amu showed the highest scavenging activity for OH• and a modest effect on ROO•, in agreement with previous records that show the potent in vitro antioxidant capacity of this extract [44].

Oxidative stress in stroke starts with the formation of the O_2_•^−^, which is produced in mitochondria as a result of the one-electron reduction of oxygen, but several additional enzymatic reactions carried by NADPH oxidases, xanthine oxidase, and others are also activated [1]; importantly, its production is enhanced during reperfusion [17]. Thus, counteracting O_2_•^−^ production with the plant extracts might possess an invaluable power against ischemia-induced damage. With respect to O_2_•^−^ scavenging activity, similar IC_50_ values were observed for the extracts from Geraniaceae, followed by those from Lamiaceae. In accord, different extracts of *Geranium* species show dose-dependent O_2_•^−^ scavenging activities, whose values are very close to those observed for quercetin. Quercetin is a flavonoid with a potent antioxidant activity [45]. Extracts of *Geranium* species have a high content of phenolic compounds; the total flavonoid content varied from 7.7 to 116.5 quercetin equivalents (mg/g), and its free radical scavenging activity may come from the presence of gallic acid derivatives [21]. Likewise, the main groups of active constituents in the Lamiaceae *Salvia* spp. are more than 20 phenolic acids and flavonoids, whose antioxidant effects include anti-lipid peroxidation and free radical scavenging. Their capacity to scavenge O_2_•^−^ is variable and depends on its specific flavonoids content [21]. Therefore, the antioxidant properties of the compounds present in the extracts might explain their O_2_•^−^ scavenging activities. 

On the other hand, the Annona extracts (Amu, Ad, Are, and Ach, with the exception of Ap) showed a very low O_2_•^−^ scavenging activity. Previous studies showed good but variable activity of both the methanol extract and water fraction of *Annona* extracts [42,46,47]. Of the 39 wild edible fruits consumed in Panama, Amu exhibited one the highest antioxidant capacity (928.57 mg Trolox^®^ equivalents/100 g fresh weight) with a high phenolic content while Ap (*Annona purpurea)* presented the lowest antioxidant activity (16.22 mg Trolox^®^ equivalents/100 g fresh weight) with a low phenolic content [48]. The species but also the processing of the plant and the method of quantification might explain the discrepancies found with our study. 

In fact, the extraction method used in this work was the maceration of powdered dry plant material in ethanol; the macerate was left for one week, and after filtration and evaporation, the extracts were obtained. This extraction procedure is the most commonly used due to its simplicity; however, the extraction time is extensive, and the yield is low [49]. Perhaps changing the extraction method would provide extracts that showed more significant effects on the measured parameters. Nonetheless, it is clear that the effects of the extracts were substantial. 

On the other hand, it has been reported that, compared with other antioxidants, O_2_•^−^ scavenging activity of RSV is high [38,50,51], However, using the xanthine–xanthine oxidase system, we observed that the antioxidant effect of RSV is related to the inhibition of the production of O_2_•^−^ and, furthermore, to its neutralization. Therefore, we could not compare the activity of the extracts with RSV. Thus, to achieve a proper comparison with RSV, the analysis of purified components of each extract must be performed.

### 4.1. Extracts Prevent Oxidative Stress Induced by Excitotoxicity in Cultured Neurons

Glutamate is the principal excitatory neurotransmitter in mammalian cells, and its excessive liberation during stroke initiates a process called excitotoxicity. Overstimulation of the glutamate N-methyl-D-aspartate type receptor results in cell death. The injury is associated with failing calcium extrusion from the cell. When calcium is accumulated in the mitochondria causes cellular damage, which has been related to oxidative stress [1]. To evaluate if the extracts have an intracellular antioxidant effect, we used an in vitro model of excitotoxicity. We found that all the extracts have cytotoxic effects at the concentration of 1 μg/mL. In contrast, when lower concentrations were used, the extracts showed different effects, which varied according to the family of the plants.

The higher concentrations of extracts from the Annonacea family showed a cytotoxic effect, a response that could be associated with the previously described anti-cancer effect of some species of this family [52]. The Ach extract was the most lethal with a concentration-dependent cytotoxic effect, followed by Are and Amu extracts, which have a high level of toxicity from 0.1 μg/mL. Previous studies using methanol and water fractions of the Are showed growth inhibition using 250 μg/mL in human hepatocellular carcinoma HepG2 cells, but lower doses (50–200 μg/mL) have a protective effect [53]. These data suggest that neuronal cultures have a high sensibility to components of the extract. In agreement, the Annonaceae family (Amu and *A. squamosa*) contain neurotoxic benzyltetrahydro-isoquinoline alkaloids that could be a central etiological factor to induce Parkinsonism [53] The development of neurodegenerative disease also has been related to the consumption of annonaceous acetogenins. The prototypical acetogenins annonacin decreased brain ATP levels by 44%, causing neuropathological abnormalities including the loss of neurons accompanied by a significant proliferation of astrocytes and microglial cells [53].

On the other hand, we found that extracts from Lamiaceiae and Geraniaceae plants have a protective effect at concentrations fluctuating from 0.01 to 0.1 μg/mL. In agreement, different ethanol extracts from *Geranium* species showed dose-dependent but negligible cytotoxicity in the concentration range of 0.1–10 μg/mL; although, water extracts of two species (*G. psilostemon* and *G. stepporum*) showed cytotoxicity at 10 μg/mL [21,54]. Specifically, *G. mexicanum* has a high content of (+)-catenin and (−) epicatechin, compounds that have shown neuroprotective effects in ischemia models [21,22]. Catechin can improve mitochondrial function and relieve apoptosis through promoting activation of AKT cell signaling [23], suggesting a mechanism that might prevent neuronal death. *Salvia* species contain phytochemicals as terpenoids, diterpenoids, and phenolic acids with pharmacological activities for the treatment of cardiovascular, immune, hepatic, and renal diseases [55] Likewise, components of *Salvia mitiorrhiza*, such as polyphenols, tanshinones, and phenolics, protect against brain disease [56]. These results indicate that the extracts with the highest level of protection against free radical species but with the lowest level of cytotoxicity to neurons could be good candidates for the treatment of diseases associated with oxidative stress in the brain. 

### 4.2. Sp Extract Protects against Cerebral Ischemia-Induced Damage

MCAO in the rat is the ischemic model that better simulates stroke in humans. In cerebral ischemia, O_2_•^−^ is the principal free radical produced and is associated with damage after reperfusion [16]. Therefore, we first tested the effect of GmPA, GnPA, Sa, and Sp on the intracellular production of O_2_•^−^ induced by glutamate exposition. As could be expected, all extracts reduced O_2_•^−^ production. However, when we tested the extracts in the in vivo model of cerebral ischemia, we observed that while Sp reduced the damage induced by reperfusion, GmPA was excessively toxic to animals. The drastic difference in the effect observed for both extracts could be associated with specific properties of individual components. Although, some components of the extracts have been isolated, and additional studies are thus necessary [21].

We postulated that compounds present on Sp extract, besides lowering the stress oxidative-induced damage, might allow adaptations on gene regulation. For example, aerial parts of Sp contain eleven neo-cleorodane difepenoids; some of these compounds augmented the expression of extracellular matrix components (e.g., genes codifying type I, II, and V collagens and elastin) [57], which are critical for vascular basement membrane function and play a central role in cerebrovascular diseases [58]. Compounds in *S. mitiorrhiza* can also inhibit the expression of adhesion molecules in vascular endothelium and in leukocytes that help to prevent the development of vascular damage induced by ischemia and reperfusion [59]. Additionally, clerodane dipertenos have been isolated from different *Salvia* species. Clerodane dipertenos activate opioid receptors [60]. The main active constituent isolated from the leaves of *S. divinorum* is the neoclerodane diterpene salvinorin A, which has shown reduced infarct volume and improved neurological deficits. It also reduced Evans blue extravasation, suggesting reduced impairment of the blood-brain barrier, and decreased the expression of cleaved casepase-3, IL-10, and TNF-alpha in the penumbral areas, preventing apoptosis and reducing inflammation [27]. Thus, our results suggest that extracts from Lamiaceae could be an alternative for treating cerebral ischemia, but it will be necessary to characterize the components of the extract; that is, the identification and evaluation of the active compounds will be required to elucidate its mechanism of action in the induced protection against cerebral ischemia

## 5. Conclusions

Our findings showed the protective effect of the extracts obtained from Annonaceae, Lamiaceae, and Geraniaceae against neuroexcitotoxicity. These results are important in the search for new treatments for diseases related to cellular stress, because they supposedly do not show harmful side effects for humans. We found that the Sp extract of the Lamiaceae family protects against neuronal damage induced by cerebral ischemia. This effect could be related to its high content of phenolic compounds and flavonoids, which are reported to have high antioxidant activity. However, it has also been reported that members of this plant family may contain high levels of terpenoids, diterpenoids, polyphenols, and phenols that also had a protective effect on the brain. We consider that one of the limitations of our study is that by using plant extracts we cannot identify the molecules that promote the protective effect in cells and their mechanism of action. Likewise, this situation limits us to say that the observed protective effect has to do with the regulation of oxidative stress caused in the cell during excitotoxicity. However, once purified the active compounds can directly connect cell signaling related to survival and protection systems in the cell, as does RSV and plant-derived antioxidants. On the other hand, we also demonstrate the cytotoxic effect of plant extracts of the Annonaceae family in this model of cellular stress. However, they can be studied to find new cancer treatments. Furthermore, if the compounds with high activity are purified, Annonaceae and Geraniaceae could be also candidates for obtaining important antioxidants for the treatment of cellular stress.

## Figures and Tables

**Table 1:** Medicinal plants investigated.

Abbreviations	Botanical Species (Family)	Voucher Specimen	Parts Used ^a^	Yield [% (w/w)]	Common Name
Ach	*Annona cherimola* Miller (Annonaceae)	15795	L	4.5	cherimoya
Ad	*Annona diversifolia* Safford (Annonaceae)	16248	L	5.9	ilama
Amu	*Annona muricata* Linn (Annonaceae)	15943	L	15.4	Soursop
Ap	*Annona purpurea* Moc & Sesse (Annonaceae)	16293	L	9.4	toreta
Are	*Annona reticulata* Linn (Annonaceae)	15944	L	5.2	custard apple
GmPA	*Geranium mexicanum* H. B & K (Geraniaceae)	14405	AP	15.5	geranium
GnPA	*Geranium niveum* S. Watson (Geraniaceae)	36899	AP	5.5	geranium
Sa	*Salvia amaríssima* Ortega (Lamiaceae)	16263	AP	3.8	sage
Sp	*Salvia polystachya* Ortega (Lamiaceae)	16386	AP	7.3	sage

*^a^* L (leaf) and AP (aerial parts).

**Table 2:** Scavenging ability (IC_50_) of plant extracts and reference compounds.

Extracts/Standards	O_2_•^−^	OH•	ROO•
**Ach**	X	155.04 ± 4.40	102.43 ± 42.92
**Ad**	X	131.39 ± 26.77	27.14 ± 8.92
**Amu**	X	98.96 ± 19.58	30.42 ± 8.00
**Ap**	23.49 ± 9.12	151.73 ± 36.54	4.48 ± 0.24
**Are**	X	126.98 ± 31.60	13.13 ± 3.67
**GmPA**	26.83 ± 10.52	147.14 ± 43.43	12.33 ± 4.26
**GnPA**	20.27 ± 4.09	131.15 ± 40.37	10.46 ± 2.47
**Sa**	46.64 ± 9.49	127.99 ± 36.85	24.91 ± 5.88
**Sp**	118.97 ± 31.98	154.18 ± 52.21	18.21 ± 3.05
**Resveratrol**	28.28 ± 9.27	30.02 ± 6.58	0.23 ± 0.06
**DMTU**		226.09 ± 45.68	

Ach, Annona cherimola; Ad, Annona diversifolia; Amu, Annona muricata; Ap, Annona purpurea; Are, Annona reticulata; GmPA, Geranium mexicanum; GnPA, Geranium niveum; Sa, Salvia amaríssima; Sp, Salvia polystachya. IC_50_ values are expressed as μg/mL. O_2_•^−^, superoxide anion; OH•, hydroxyl radicals; ROO•, peroxyl radical; DMTU, dimethylthiourea. X, the IC_50_ was not reached. Data are mean ± SD; *n* = 3–4.
